# Two-month longitudinal study of mechanical properties of absorbable sutures used in orthopedic surgery

**DOI:** 10.1186/s13018-016-0451-5

**Published:** 2016-10-12

**Authors:** Daniel A Müller, Jess G Snedeker, Dominik C Meyer

**Affiliations:** Department of Orthopedics, University Hospital Balgrist, Forchstrasse 340, 8008 Zürich, Switzerland

**Keywords:** Suture, Absorbable fiber, Material properties, Degradation, In vitro, Orthopedic surgery

## Abstract

**Background:**

This is the first study assessing the properties of large-diameter degradable sutures relevant for orthopedic applications over the course of in vitro incubation for 2 months. The data we present here provide guidance to the orthopedic surgeon in predicting the long-term performance of suture materials used everyday in surgical practice.

**Methods:**

Five different absorbable (Vicryl, Maxon, Monocryl, PDS II, Vicryl rapide) and one non-absorbable (Ethibond) suture materials were tested. Measurements were made at five time points during the 56 days of incubation under physiological conditions (37.0 ± 0.02 °C; pH 7.4 ± 0.2). The following variables were recorded: load to failure, strain at maximal load as elongation normalized to original length, stiffness as the ratio of load to displacement on the linear proportion of the stress strain curve, and hysteresis as area under the curve of the stress strain curve.

**Results:**

Vicryl was the strongest fiber on day 0 (195 N); however, by day 42, the tensile strength of the suture reduced to 14 N. Between days 14 and 28, PDS II (171 N) and Maxon (182 N) sustained the highest loads. Monocryl (*p* = 0.003) and Maxon (*p* < 0.001) showed an increasing strain with time, whereas Vicryl (*p* = 0.002) and Vicryl rapide (*p* = 0.007) revealed an increasing material stiffness. Furthermore, both Vicryl (*p* = 0.053) and Monocryl (*p* < 0.001) had an increasing hysteresis with ongoing degradation. Maxon, PDS II, and Ethibond showed stable material properties during the 2 months.

**Conclusions:**

The three absorbable sutures Vicryl, PDS II, and Maxon could sustain higher loads during the first 2 weeks than the non-absorbable Ethibond. Unexpectedly, Maxon and PDS II maintained their elastic properties in spite of their proceeding degradation and loss of tensile strength.

## Background

Medical suture material is essential to appropriately position and hold tissue until healing has occurred. Particularly in orthopedic surgery, the suture material may be subjected to considerable mechanical loads for a long time period, as healing after tendon, ligament, or fascia repair takes place over two or more postoperative months.

Because the suture is often no longer needed after healing, different biodegradable suture materials have been developed and are in widespread surgical use. The materials used in such sutures can differ not only in tensile properties like strength and stiffness but also in knot strength. Data on longitudinal retention of mechanical strength is usually available over a limited time frame from the suture manufacturer. Yet, a thorough description of the suture’s mechanical behavior (e.g., stiffness, hysteresis) over a time frame relevant to musculoskeletal injuries has not been reported, despite the fact that substantial changes of these properties with suture degradation is certain [1, 2].

The speed of suture degradation caused by hydrolysis depends not only on the material but also on the fiber diameter, tissue temperature, and pH [3–5]. Biodegradation of absorbable sutures has been studied in the discipline of visceral surgery, whereas such fibers are too weak for orthopedic purposes [6–10]. There is no data available regarding time-dependent mechanical properties of absorbable sutures for large-diameter fibers including sizes United States Pharmacopeia (USP) 0 and more. The existing data remains limited regarding the influence of time or focuses on highly specific orthopedic applications involving arthroscopic surgery and bone anchors [11–16].

Therefore, we set out to characterize how (1) failure load, (2) failure strain, (3) suture stiffness, and (4) suture hysteresis of six different suture materials would vary over the course of in vitro incubation for 2 months. We hypothesized that longitudinal changes in suture mechanical properties would be highly material dependent and possibly more important the pure development of failure load as stated by the manufacturers.

## Methods

### Sutures

**Fig. 1:**
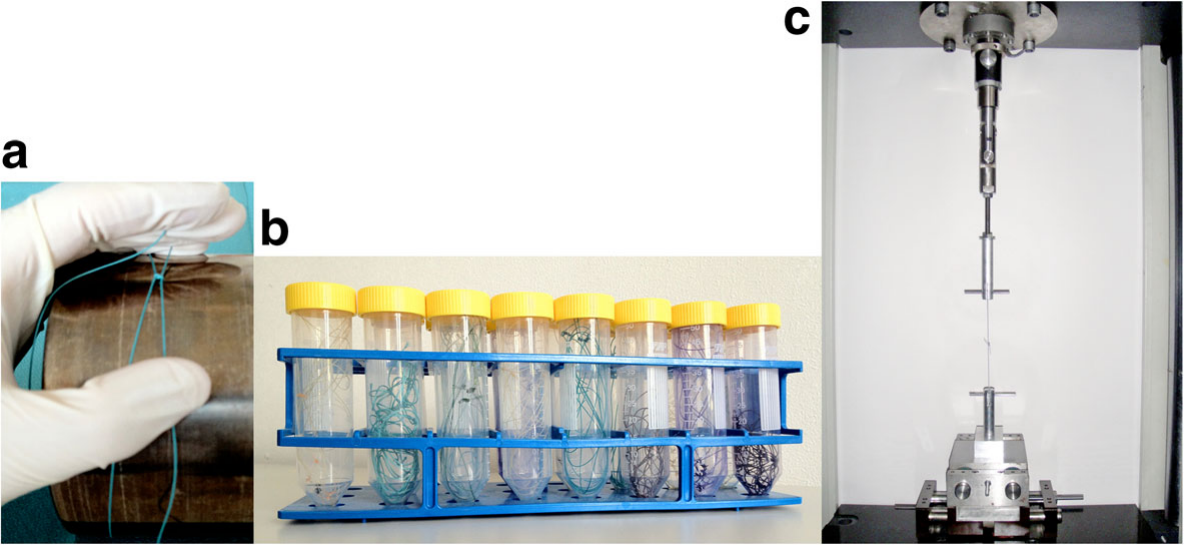
Preparation, incubation and testing of the suture loops. **a** Creating the suture loop by knotting around a metallic cylinder. **b** Six loops of the same suture material are incubated together in a polyethylene tube containing 50 ml of an approved standard testing solution. **c** Fiber loop fixed in material testing machine over a metal bar on each side

We tested five different absorbable and one non-absorbable suture materials that are used daily in orthopedic surgery (see Table 1). All the sutures were chosen with a diameter of USP 1, being an appropriate size for suture of fascia, capsule, and tendons. Furthermore, it is the largest diameter in every tested suture material that is available. In preparation for testing, the needle was removed and the sutures were circled around a metallic cylinder with a diameter of 12 cm (see Fig. 1a). Each suture loop was tied with five standard surgical square knots. A single surgeon tied all the knots. From each suture type, 60 loops were produced in the described matter.

**Table 1:** Suture properties

Suture	USP	Material		Bioabsorbability	Manufacturer
Vicryl	1	Poly/-lactide-coglycolide	Braided	Aborbable	Ethicon
PDS II	1	Polydioxanone	Monofilament	Absorbable	Ethicon
Maxon	1	Polyglycolide-cotrimethylene carbonate	Monofilament	Absorbable	Syneture
Monocryl	1	Polyglecaprone 25	Monofilament	Absorbable	Ethicon
Vicryl rapide	1	Poly/-lactide-coglycolide	Braided	Absorbable	Ethicon
Ethibond	1	Poly/-ethylene terephthalate	Braided	Non-absorbable	Ethicon

### Incubation and fluid

Measurements were made at five time points corresponding to 0, 14, 28, 42, and 56 days of incubation under physiological conditions. All suture materials were incubated together in a polyethylene tube containing 50 ml of a standard testing solution for absorbable materials (see Fig. 1b) [17]. The used solution is approved by ASTM (American Society for Testing and Materials) for testing absorbable sutures. The solution components correspond to ringer solution. As this solution is standardized, it provides reliable comparison of testing results to other mechanical studies of absorbable sutures. All tubes were then kept at a constant temperature of 37.0 ± 0.02 °C. The pH of the solution was buffered to 7.4 ± 0.2 for the whole time of incubation, and the value was controlled before each testing time point.

### Measurements

Measurements on 12 loops were made for each material and at each time point. Samples from each suture material were taken out of the incubator at the appropriate time. Samples were divided into two groups of six loops each of the same material. The loops were fixed in a material testing machine (Zwick1456, Zwick GmbH, Ulm, Germany) as shown in Fig. 1c. The loops were mounted over two metal bars which are moved apart by the machine creating a stress to the fiber.

Using the first group of sutures, load to failure was recorded. For this purpose, loops were pre-tensioned to 1 N and loaded until failure at a rate of 10 mm/s. The second group was used to evaluate the material properties under a repetitive load. After positioning in the testing machine, the loops were pre-tensioned to 1 N. Thirty cycles of load ranging from 1 N to 50 % of the mean load to failure (as measured in the first group) was applied. The loop was then ramp loaded until failure at 10 mm/s. All force displacement data were digitally recorded using the test machine software.

As suture loops (suture/knot construct) were tested in this experiment, the obtained results reflect properties of both suture material and knot security. For this reason, we marked the fiber on each side of the knot to control for knot tightening during the tests. The elongation distance through knot tightening was in all loops insignificant compared to the material elongation.

### Analyzing data and statistics

The following variables were recorded for each suture sample: (1) load to failure with and without cyclical loading, (2) elongation of the fiber at maximum load, normalized as the ratio of the end length to the initial loop length, (3) stiffness as the slope of the force/displacement curve in a linear region of the material curve, and (4) hysteresis as the non-elastic energy loss during a cycle of loading and unloading, here normalized as a percentage.

Commercial statistical software (Stata v12.1, StataCorp.) was used for all analyses. Recorded values are indicated as mean with their standard error (mean ± standard error). Graphical results indicate 95 % confidence intervals. A one-way ANOVA was used first to identify differences among the different suture materials, with post hoc analysis of group means using unpaired Student’s *t* test. The level of significance was set at 0.05.

## Results

### Load to failure

On day 0, before any incubation, the Vicryl suture showed the highest load to failure (195 ± 4 N). PDS (145 ± 3 N) and Ethibond (145.7 ± 2 N) were on a similar level with no significant difference. Interestingly, the two absorbable fibers Vicryl (195 ± 4 N; *p* < 0.001) and Maxon (164 ± 7 N; *p* = 0.036) were both stronger than the non-absorbable Ethibond. The lowest load was detected on Vicryl rapide (51.0 ± 0.9 N).

**Fig. 2:**
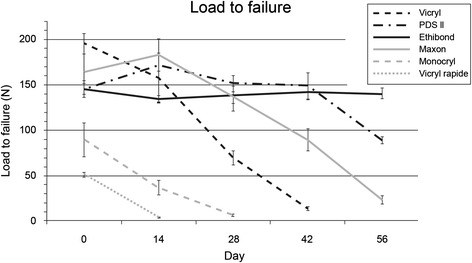
Load to failure. Load to failure in Newton on different time points (mean, upper 95 % CI and lower 95 % CI)

The development of the load to failure during the 2 months of incubation was characterized (Fig. 2). Even on day 14, the absorbable sutures Vicryl (157 ± 9 N; *p* = 0.049) and Maxon (183 ± 6 N; *p* = 0.001) sustained higher loads than the non-absorbable Ethibond (135 ± 1 N), with the Vicryl loops showing the fastest loss of load bearing capacity. After day 42, a conclusive evaluation of Vicryl loops was impossible as the loop was too weak for support the 1-N pre-load. PDS (151 ± 3 N) and Maxon (137 ± 6 N) featured comparable rates of mechanical degradation through day 28, after which the strength of Maxon loops decreased more rapidly (*p* < 0.001). As expected, the non-absorbable Ethibond loops showed no significant differences over time and had the highest load bearing capacity at the final measurement point (140 ± 2 N). The Monocryl suture was no longer able to sustain the preload of 1 N at day 28, while Vicryl rapide was unable to support load already at day 14.

After applying the cyclic load, the suture loops were again loaded until they failed. In general, there was no significant difference between the load to failure with or without cyclic loading. Exceptions were PDS on day 0 (145 vs. 121 N; *p* < 0.001) and Maxon at day 14 (183 vs. 155 N; *p* = 0.044). Ethibond showed a significant difference in ultimate load after cyclical loading at three time points, namely day 0 (146 vs. 134 N; *p* = 0.04), day 42 (141 vs. 131 N; *p* = 0.028), and day 56 (140 vs. 126 N; *p* = 0.028).

### Strain at maximal load

**Fig. 3:**
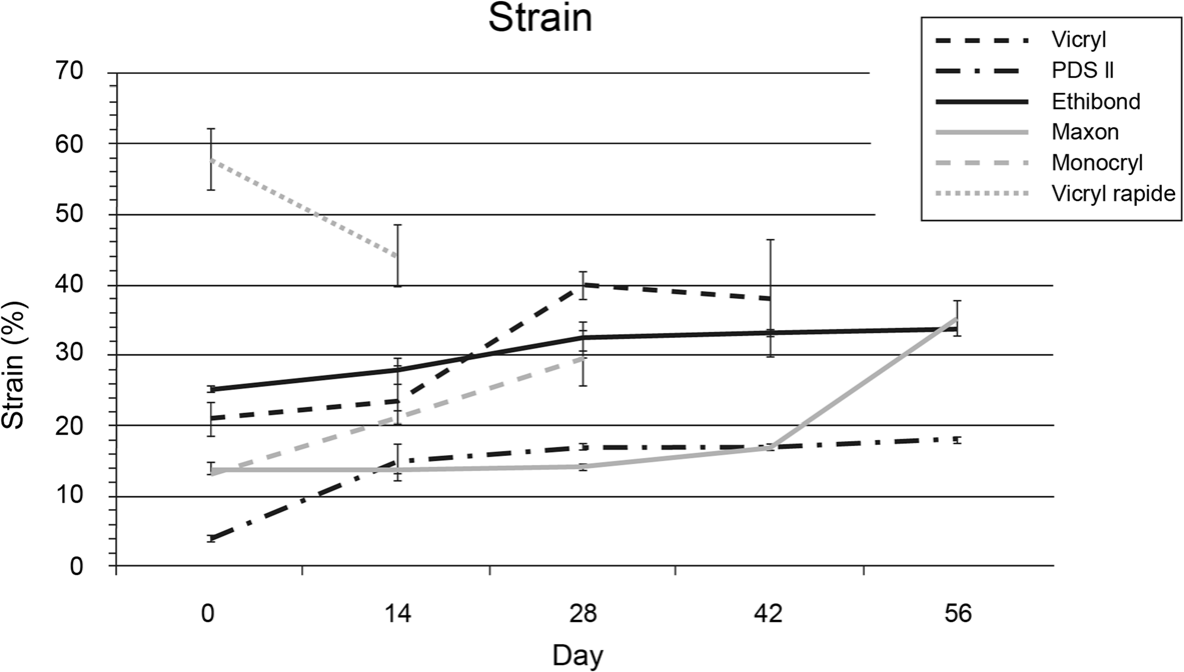
Strain at maximal load after cyclic loading. Strain at maximal load as elongation normalized to original length, on different time points (mean, upper 95 % CI and lower 95 % CI)

The maximal strain of the loop at maximal load on day 0 was highest in Vicryl rapide (57 ± 1.6 %). In PDS and Ethibond, the course was stable. Monocryl (from 13.1 to 30.0 %; *p* = 0.003) and Maxon (from 14.2 to 35.2 %; *p* < 0.001) showed an increasing maximal strain with ongoing time (see Fig. 3).

### Stiffness

**Fig. 4:**
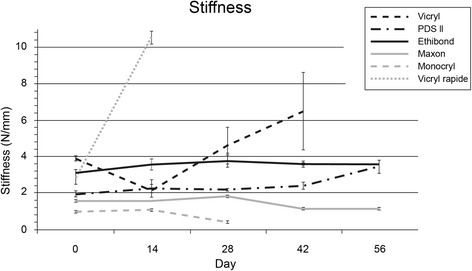
Stiffness after cyclic loading. Stiffness as the ratio of load to displacement on the linear proportion of the stress strain curve, on different time points (mean, upper 95 % CI and lower 95 % CI)

Both Vicryl (from 3.89 to 6.49 N/mm; *p* = 0.002) and Vicryl rapide (from 2.84 to 10.5 N/mm; *p* = 0.007) revealed an increasing stiffness over time (see Fig. 4). Maxon and Ethibond had stable stiffness values over the 2-month test period without significant differences between the measurement points. PDS showed constant values until day 42; afterwards, the stiffness increased (from 2.40 to 3.44 N/mm; *p* = 0.014). Monocryl on the other hand had a decreasing stiffness between day 14 and 28 (from 1.09 to 0.42 N/mm; *p* < 0.001).

### Hysteresis

**Fig. 5:**
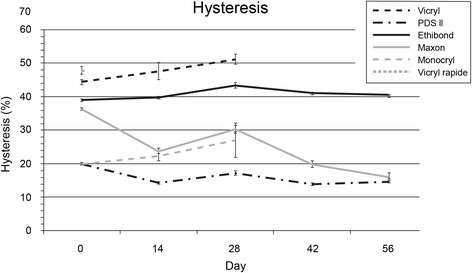
Hysteresis after cyclic loading. Hysteresis as area under the curve of the stress strain curve (representing the mechanical work absorbed by the suture material during testing), for simplification described as ratio to the area under the curve of the first half of the cyclic load, on different time points (mean, upper 95 % CI and lower 95 % CI)

As in the measurements of maximal strain, PDS and Ethibond had constant hysteresis values over the time course (see Fig. 5). The Maxon loop revealed decreasing hysteresis (from 36.3 to 15.9 %; *p* = 0.004). In contrast, both Vicryl (from 47.6 to 51.2 %; *p* = 0.053) and Monocryl (from 20.1 to 27.0 %; *p* < 0.001) had increasing hysteresis with ongoing degradation.

## Discussion

Soft tissue such as the tendons, fascia, ligaments, or skin are often surgically positioned and fixed using surgical suture material. In comparison to other surgical specialties, orthopedic surgery features particularly forces with repetitive loading on the repair site that can jeopardize appropriate healing. While suture retention in the tissue is one major issue, failure of the suture itself is common. Further, the act of seating the stitch and knot may lead to suture loop elongation and gap formation, events that may also lead to compromised healing [18]. These factors should be considered by the surgeon when choosing an appropriate suture material, particularly with regard to load bearing capacity and elasticity of the suture material.

Numerous choices for a suture material are available, including biodegradable sutures. Biodegradable material can be viewed as well suited for applications in which the suture is no longer required after healing has occurred. However, for repairs of tissues that are subjected to high mechanical demands, degradable sutures are often not utilized by the surgeon. This has mainly to do with uncertainty regarding the expected duty life of the sutures related to their progressive hydrolysis and corresponding loss of mechanical integrity. While several studies have described the biomechanical properties of large diameter surgical sutures in various conditions, these studies neglect the effect of time [12, 14, 19, 20].

Therefore, the aim of this study was to provide data on the mechanical properties of the most common suture materials used in orthopedics. In particular, the changes of the material properties during an observation period of 56 days were investigated.

One limitation of this study was the use of an in vitro conditioning of the materials that did not comprise cyclic load. Such loads can be expected to result in material creep, and how this effect may influence the long-term properties of the sutures may be considered in future tests. In any case, there were no significant differences in load to failure with and without cyclic load. The environmental factors during the incubation time (pH 7.4 and temperature 37 ° C) were standardized using an approved standard testing solution to simulate appropriate values as in vivo. Regarding the here investigated mechanical properties, we are confident that using standardized in vitro conditions simulating the wet and warm milieu of the body reproduces the in vivo conditions adequately. Using an in vivo model would be useful to test the combined development of tissue and suture, but would be less useful to test the development of the suture properties alone.

We confirmed that Vicryl rapide and Monocryl lose their mechanical strength relatively quickly, with an effective loss of integrity after an incubation period of 14 and 28 days, respectively. Due to the early degradation, other properties including maximal strain and stiffness also showed significant changes in the first weeks. These materials appear to be only suitable for situations in which rapid healing is expected, with minimal longer-term mechanical demands. This would concern applications such as in ligations or skin closure, where for example subcutaneous irritation makes early absorption desirable.

Regarding the consequences on the best choice of suture material for the orthopedic surgeon, of course the final decision is to be made by individual surgeon. However, in the case of degrading sutures, there is a race between increasing strength of the developing scar and decrease of the sutures properties. Scar healing occurs with a speed of roughly 25 % at 6 weeks, 45 % at 12 weeks, and 80 % at 24 weeks of the final strength [2]. Therefore, the suture should correspondingly preserve 75, 55, and 20 % at the according time points. Particularly, the elasticity of the suture may be beneficial to compensate for sudden but rare spikes in loading on the suture-repair construct, in order to prevent failure or breakage. We could show that particularly PDS will preserve its advantageous properties over the time of absorption, with corresponding lower maximum tensile strength.

The three absorbable sutures Vicryl, PDS, and Maxon all could sustain higher loads during the first 2 weeks than the non-absorbable Ethibond. As expected, the properties of the non-absorbable Ethibond did not differ over time. Unexpectedly, also Maxon and PDS maintained their elastic properties in spite of their proceeding degradation and loss of tensile strength. Thus, even after considerable loss of failure strength, the relative elasticity of these materials remained intact and the suture could still be expected to adequately support transient tissue loads. However, while PDS may be considered useful to bear clinically relevant loads on the tendon, ligaments, or bone, Maxon and Vicryl may be rather considered where mechanically less challenged adaptation of tissue is performed such as in closure of peritendineum, side-to-side repairs of fascia, and similar situations.

## Conclusions

In summary, this is the first study assessing the properties of large-diameter degradable sutures during a time period relevant for orthopedic applications. The non-absorbable Ethibond and surprisingly the absorbable Maxon and PDS revealed relatively constant values despite the process of degradation.

The choice of an appropriate suture material should consider the expected duty loads in context of tissue healing time. The data we present provides some guidance to the orthopedic surgeon in better predicting the long-term performance of suture materials used everyday in surgical practice.

## Abbreviations

CI: Confidence interval
USP: United States Pharmacopeia
